# Artificial Synaptic Devices Based on Natural Chicken Albumen Coupled Electric-Double-Layer Transistors

**DOI:** 10.1038/srep23578

**Published:** 2016-03-24

**Authors:** Guodong Wu, Ping Feng, Xiang Wan, Liqiang Zhu, Yi Shi, Qing Wan

**Affiliations:** 1 School of Electronic Science and Engineering, and Collaborative Innovation Center of Advanced Microstructures, Nanjing University, Nanjing 210093, China

## Abstract

Recent progress in using biomaterials to fabricate functional electronics has got growing attention for the new generation of environmentally friendly and biocompatible electronic devices. As a kind of biological material with rich source, proteins are essential natural component of all organisms. At the same time, artificial synaptic devices are of great significance for neuromorphic systems because they can emulate the signal process and memory behaviors of biological synapses. In this report, natural chicken albumen with high proton conductivity was used as the coupling electrolyte film for organic/inorganic hybrid synaptic devices fabrication. Some important synaptic functions including paired-pulse facilitation, dynamic filtering, short-term to long-term memory transition and spatial summation and shunting inhibition were successfully mimicked. Our results are very interesting for biological friendly artificial neuron networks and neuromorphic systems.

Recently, bionic devices have gained considerable attention for their ability to mimic and explore superior functions of biological systems^1^,^2^,^3^. Among these bio-inspired devices, artificial synapses are of great significance for neuromorphic systems because they can emulate the signal process and memory functions of biological synapses^4^. Conventionally, silicon-based integrated circuits have been employed as artificial synapses and neurons, but these circuits consumed considerably more energy than biological synapses^5^. Hence, it is essential to develop scalable and low-power devices to scale neuromorphic system towards the level of the human brain. These demands have prompted two-terminal memristors as promising candidates for artificial synapses because they can be implemented by silicon-compatible technology with high density and emulate synaptic plasticity with low power consumption^6^,^7^,^8^. In order to obtain wider support for artificial synapse research from the viewpoint of devices and materials, three-terminal electric-double-layer (EDL) transistors were also creatively designed for artificial synapse applications^9^,^10^. For example, excitatory postsynaptic current, dynamic logic, learning and memory functions of biological synapses were successfully mimicked in carbon nanotube transistors gated by proton-incorporated polymer dielectrics^11^. Ion-related EDL modulation and interface electrochemical doping under different pulse voltages are the primary mechanisms for the short-term and long-term plasticity, respectively^12^. The emergency of these new-concept synaptic transistors greatly enriched the scope of artificial synapses in materials and devices.

Meanwhile, recent progresses in using natural biomaterials to construct functional electronic devices have received growing attention for the new generation of environmental friendly and biocompatible electronic systems^13^,^14^,^15^. As a kind of biological material that is widely available, proteins are essential natural component of all organisms. In the past few years, proteins were generally employed to fabricate functional electronic devices, such as field-effect transistors, resistive switching memory and diodes^16^,^17^,^18^,^19^,^20^. In this report, natural chicken albumen films with high proton conductivity were used as the electrolyte dielectrics for indium-zinc-oxide (IZO) synaptic transistor fabrication. Important synaptic functions including paired-pulse facilitation, dynamic filtering, short-term to long-term memory transition, and spatial summation/shunting inhibition were successfully mimicked. Our results are interesting for biocompatible artificial neuron networks and neuromorphic systems.

## Results and Discussion

**Figure 1:**
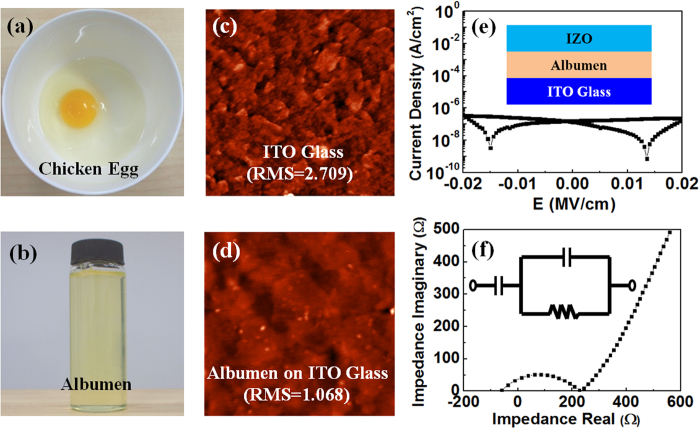
(**a**) Optical image of a broken chicken egg in a bowl (including egg white and egg yolk) (Taken by Dr. Guodong Wu). (**b**) Thenatual albumen (egg white) was seperated and stored in a glass bottle (Taken by Dr. Guodong Wu). AFM images of (**c**) ITO glass substrate and (**d**) albumen film spin-coated on the ITO glass substrate (area: 3.0 μm × 3.0 μm). (**e**) Leakage current density characteristics of the albumen film measured in an ITO/albumen/IZO sandwiched structure shown in the inset (Drawn by Dr. Guodong Wu). (**f**) A typical Nyquist plot ofthe albumen film. The inset is thediagram of the equivalent circuit used to analyze the impedance data.

Fig. 1a shows a broken chicken egg put in a bowl. The albumen liquid was then collected in a glass bottle without any subsequent treatment (Fig. 1b). The albumen liquid was then coated on the substrates by spin coating for subsequent use. Albumen, also called egg white, consists primarily of ~90% water and ~10% proteins (including predominantly of albumins and a small fraction of mucoproteins and globumins). Water solubility, emulsifying ability and ionic conductivity of chicken albumen are remarkably good ascribed to various hydrophilic functional groups such as -COOH, -NH_2_, -SH and -OH^21^. The ionic conductivity was mainly due to the presence of water, which facilitated proton transport, as demonstrated by Darvishi *et al*.^22^. However, when albumen film is heated to above 80 °C, the amino acid chains undergo a non-reversible reaction and get unfolded or uncurled (termed asdenaturation), and the ionic conductivity and specific gate capacitance will be greatly reduced^23^,^24^. In our experiment, it is necessary to maintain certain hydrophilicity and ionic conductivity for the fabrication of synaptic transistors. So, the spin-coated albumen films were only dried in the air ambient without any thermal reaction treatment. Fig. 1c,d show the AFM images (3.0 μm × 3.0 μm) of the ITO glass substrate before and after albumen coating, with a root mean square (RMS) roughness of 2.70 nm and 1.06 nm, respectively. The results indicate that the spin-coated albumen film has a very smooth surface, which is favorable for synaptic device fabrication. The smooth surface could be attributed to self-filling induced by free movement of the protein macromolecular chains^25^.

To confirm the as-prepared albumen film is an electron insulating, but ionic conducting electrolyte film, electrical property and electrochemical impedance spectroscopy (EIS) were measured^26^. Fig. 1e shows the leakage current density of the albumen film at direct current (DC) condition tested using an ITO/albumen/IZO sandwich structure (Fig. 1e, inset). The albumen film has a maximum leakage current density of 5 × 10^−7^ A/cm^2^ at −0.02 MV/cm. Such leakage current is larger than that of the traditional high-κ dielectrics, but it is still lower than that of the ionic liquids and polymer electrolytes^27^,^28^. Next, we applied alternating current (AC) potentials of 0.02 V to the albumen film and depicted the real and imaginary parts of the impedance in Nyquist plots (Fig. 1f). A semicircle in the high-frequency region and an inclined spur in the low-frequency region can be observed. Such curves are fingerprints of ion conductors contacted by blocking electrodes, with the semicircle corresponding to the bulk ionic impedance and the inclined spur to the pile-up of ions at the electrodes^29^. The curves can be fitted by a simple equivalent circuit (Fig. 1f, inset) which has been used to model ionic conductor film accurately by accounting for the bulk impedance and capacitive effect at the contacts. This model is applicable in our albumen film and the equivalent circuit yields a bulk resistance which can be translated into an proton conductivity of ~3.7 × 10^–4^ S/cm^30^. The results indicate that the albumen film is a good ion conducting film.

In chemistry, the majority component (>75%) of protein in albumen is albumin with a p*K*a value of 4.7^31^. Since H_2_O can interact with amino acidresidues in albumin, mobile ions can be generated in hydrated albumen. In atmosphere environment, hydrated albumen can be treated as an aqueous solution locally at the molecular level. The amino acid interact with H_2_O and dissociate into negatively-charged (CH_2_)_2_-COO^−^ side chains and H^+^ (H_3_O^+^) ions^32^. The ion conducting phenomenon in the albumen film is mainly attributed to the migration of protons. In other words, the albumen film here is a proton conducting film. Here, we should point out that water absorption also creates plenty of proton-conducting hydrogen-bond chains which can serve as proton wires for proton migration, and protons can move along the hydrogen-bond network following the Grotthum mechanism^33^. Abundant mobile protons are essential for high proton conductivity in our albumen proton conductor film.

**Figure 2:**
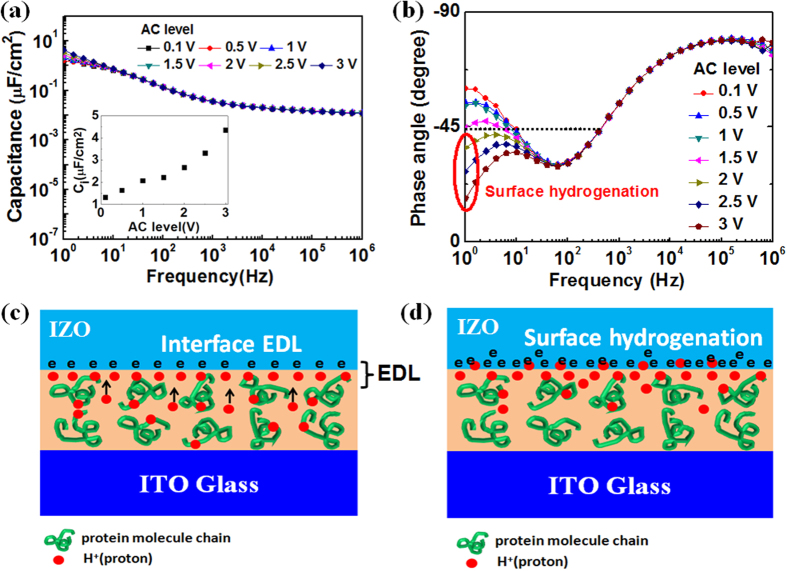
(**a**) Specific capacitance and (**b**) phase angle of the albumen film as a function of frequency under different AC potentials. (**c**) Schematic diagram of IZO/albumen interface EDL formation under low bias potential (Drawn by Dr. Guodong Wu). (**d**) Schematic diagram of IZO/albumen interface hydrogenation process under high bias potential (Drawn by Dr. Guodong Wu).

To further understand proton migration under an electric field, the specific capacitance and phase angle of the albumen film as a function of frequency under different AC potentials were characterized by EIS. Fig. 2a is the specific capacitance curve. For AC potentials from 0.1 to 3.0 V, the curves are almost coincide in the frequency region above 30 Hz and branched below 30 Hz. The specific capacitance increases with decreasing the frequency, and reaches to the maximum value (>1.0 μF/cm^2^) at 1.0 Hz. These results are mainly attributed to proton migration at high frequency and the formation of huge interface EDL capacitance at low frequency, as shown in Fig. 2c. The capacitance value at 1.0 Hz versus AC potential is plotted in the inset of Fig. 2a. When the AC potential changes from 0.1 to 3.0 V, the specific capacitance increases from 1.32 to 4.36 μF/cm^2^. At higher voltage, more protons will migrate to the interface region, resulting in a much larger interface capacitance^34^. However, at high frequency, there are no enough response time for protons to migrate to the interface and can’t contribute to the specific capacitance. Thus the curves are almost coincident when frequency is higher than 30 Hz. Fig. 2b is the phase angle curves of the albumen film as a function of the frequency under different AC potentials. The phase angle is −90^o^ for an ideal capacitor and 0 for an ideal resistor^35^. The curves indicate that the albumen film exhibits capacitive behavior more when the frequency is higher than 300 Hz (θ<−45^o^) and resistive behavior more when the frequency is between 10 and 300 Hz (θ>−45^o^). When the frequency is lower than 10 Hz, the curves begin to branch depending on the AC levels. The phenomenon is attributed to a large EDL capacitance at the albumen/IZO interface. At an AC level of >2.0 V and a frequency of <10 Hz, the mobile protons penetrate across the albumen/IZO interface and react with IZO under high voltage bias, a surface hydrogenation and reversible electrochemical process can occur (Fig. 2d). The surface hydrogenation process results in increasing of IZO conductance^36^. The mechanisms of proton migration under different electric fields are the basic principle of realizing biological synaptic short-term and long-term plasticity in the as-fabricated synaptic transistor gated by proton conducting albumen film.

**Figure 3:**
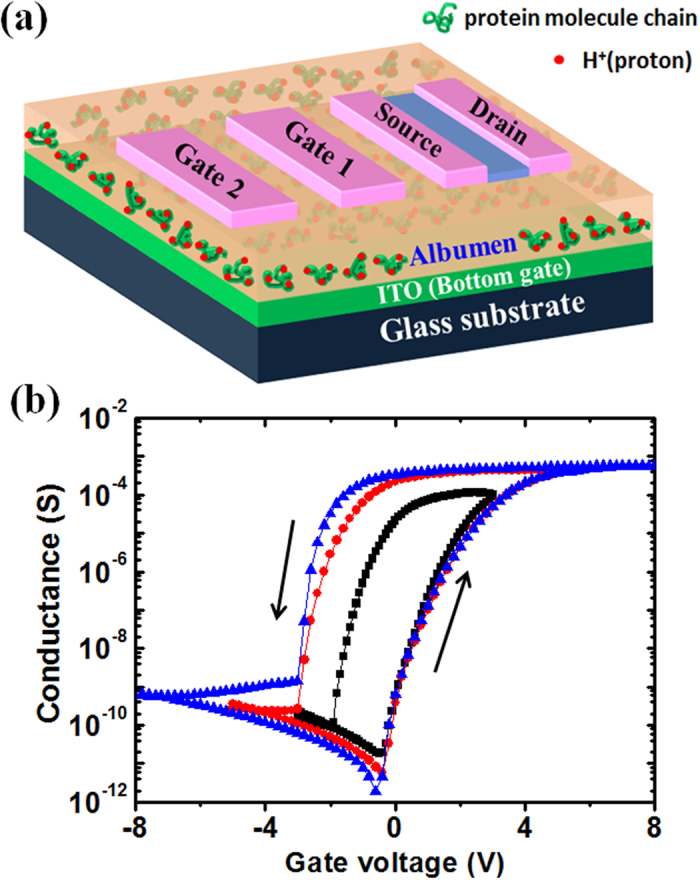
(**a**) Schematic diagram of the albumen-gated IZO-based synaptic transistor with two in-plane gates (Drawn by Dr. Guodong Wu). (**b**) Ttransfer curves (I_DS_–V_GS_) measured at V_DS_ = 2.5 V with different sweep ranges of V_GS_. Clear counter-clockwise hysteresis windows are observed during each V_GS_ sweep.

Fig. 3a shows the schematic illustration of the albumen-gated synaptic transistor with two in-plane gates. In such device, two in-plane gates (G_1_ and G_2_) and source/drain electrodes are all in the same plane, and G_1_ and G_2_ are capacitively coupled to the IZO channel layer with two gate capacitors in series through the bottom ITO floating gate. The feature of this configuration is that the synaptic device can further form a connected network with more in-plane gates through strongly lateral EDL coupling. Fig. 3b shows the transfer curves (I_DS_–V_GS_) measured at V_DS_ = 2.5 V with different bottom gate voltage (V_GS_) sweeping ranges at a sweeping rate of 200 mV/s. Clear counter-clockwise hysteresis windows are observed during each V_GS_ sweep. The hysteresis window expands with increasing the sweeping voltage range, and a maximal hysteresis window of 4.0 V can be obtained when the sweeping voltage is increased to 8.0 V. Non-volatile memory can be observed at a large gate voltage sweeping range because protons can penetrate into the IZO channel layer and induce electrochemical doping when V_GS_ is increased from 3.0 to 8.0 V.

**Figure 4:**
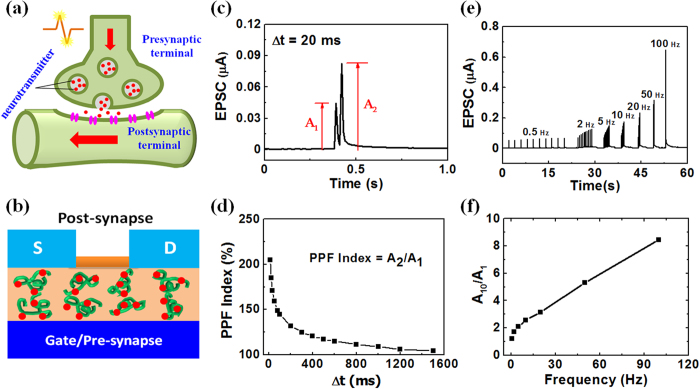
(**a**) A schematic diagram of a biological synapse (Drawn by Dr. Guodong Wu). (**b**) A schematic diagram ofour albumen-gated synaptic transistor (Drawn by Dr. Guodong Wu). (**c**) Paired-pulse facilitation. A pair of pre-synaptic spikes and the triggered EPSC under an inter-spike interval of 20 ms. (**d**) PPF index, defined as the ratio of A_2_/A_1_, plotted as a function of inter-spike interval, Δt_pre_, between the two consecutive spikes. (**e**) Dynamic filter behaviors of the albumen-gated synaptic transistor, EPSCs recorded in response to stimulus train with different frequencies (0.5 to100 Hz). (**f**) EPSC amplitude gain (A_10_/A_1_) plotted as a function of the pre-synaptic spike frequency.

In our brain, signal processing, memory and learning behaviors are achieved by modulating ion flows in the neurons and synapses. As schematically shown in Fig. 4a, ionic fluxes through the ion channel in synapses result in the movement of neurotransmitters, which can modulate synapse efficacy in establishing the relationship between the presynaptic and the postsynaptic neurons^37^. The ionic excitatory postsynaptic potential (EPSP), the basic representation of synaptic strength, is a temporary current caused by the flow of ions into the postsynaptic neuron as a result of the pre-synaptic neuron spike^38^. Similar to the biological synapses, the ITO bottom-gate electrode and IZO source/drain electrodes with channel layer are regarded as the presynaptic and postsynaptic terminals, respectively (Fig. 4b). The albumen film and ions (protons) are regarded as the synaptic cleft and neurotransmitters, respectively. The channel current (I_c_) is regarded as the excitatory postsynaptic current (EPSC). Here, EPSC is used to represent the synaptic strength instead of EPSP in order to facilitate the measurements. Proton migration triggered by presynaptic spikes applied on the gate electrode results in the change of channel current. The relationship between ITO gate electrode and IZO source/drain electrodes with channel layer is thus established and can be precisely adjusted by the concentration of protonsat the albumen/channel interface. Such a process is similar to the spike-modulated movement of the neurotransmitters in biological synapses.

Synaptic plasticity is an important neurochemical foundation of learning and memory in neuroscience, which is believed to occur in human brain as a result of short-term plasticity (STP) and long-term plasticity (LTP)^39^. Paired-pulse facilitation (PPF) is a STP process in biological systems, which is essential to decode temporal information in auditory or visual signals. When a synapse receives two presynaptic spikes in a short time, the second postsynaptic response will be larger than the first one. A higher PPF can be obtained when the inter-spike duration is reduced^40^,^41^. The PPF in our synaptic transistor is measured by applying two successive pre-synaptic spikes (0.5 V, 10 ms) with an inter-spike interval, Δt_pre_, ranging between 10 and 1500 ms. As shown in Fig. 4c, the EPSC triggered by the second pre-synaptic spike is larger than that triggered by the first spike with an inter-spike time of 20 ms. PPF index is defined as the ratio of the peak current amplitude between the second and first EPSC and plotted versus Δt_pre_, as shown in Fig. 4d. The PPF index reaches the maximum value of ~205% at Δt_pre_ = 10 ms and it decreases gradually with increasing Δt_pre_. The results indicate that PPF-related short-term synaptic plasticity is successfully mimicked in our albumen-gated synaptic transistor.

Synapses can also act as the dynamic filters for spiking information transmission depending on the signal frequency. Short-term synaptic depression contributes to low-pass temporal filtering and short-term synaptic facilitation contributes to high-pass temporal filtering^42^,^43^.In our albumen-gated synaptic transistor, temporal high-pass filtering function can be mimicked by applying a spike train with 10 voltage spikes (0.5 V, 10 ms) at each frequency on the bottom ITO gate electrode. Fig. 4e shows the EPSC responses to the spike train at different frequencies. At the frequency of 0.5 Hz, the peak value of the EPSCs is measured to be ~50 nA even after 10 voltage spikes. With increasing the frequency, the peak value of the EPSCs increases obviously. The frequency dependent gain is defined as the ratio of the amplitudes between the tenth and the first EPSC. The gain is plotted against the spike frequency in Fig. 4f. It increases from ~1.0 to ~8.5 when the spike frequency is changed from 0.5 to 100 Hz, indicating that the albumen-gated synaptic transistor can act as a dynamic high-pass filter for information transmission. The PPF and high-pass filtering emulation results are attributed to the change of channel conductance modulated by proton migration temporally. Under a low gate bias condition, after the first spike, the protons in the albumen proton conductor would drift back to the equilibrium positions due to the concentration gradient. If the interval of the next spike is very short, the protons triggered by the first spike still partially reside near the IZO channel. Therefore, the protons triggered by the next spike can be augmented by the residual protons triggered by the first spike. For a longer spike interval, there is less residual proton near the IZO channel, which will result in a lower PPF value.

**Figure 5:**
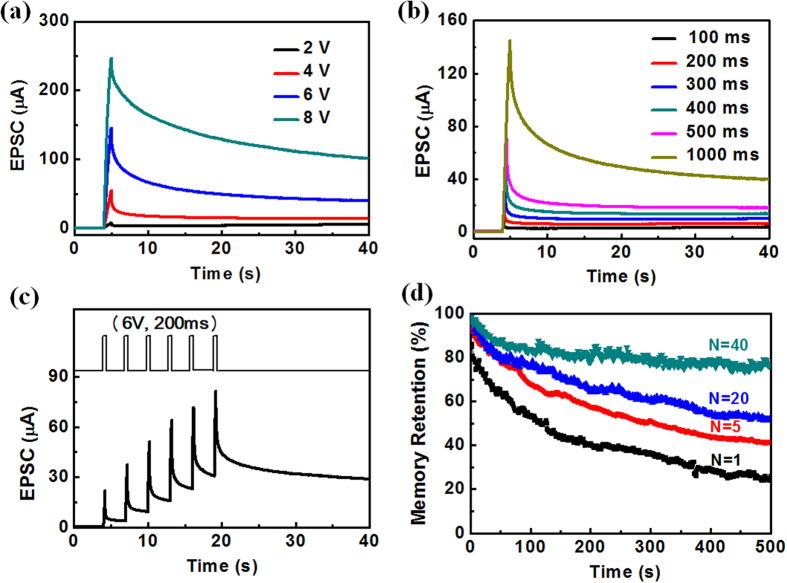
(**a**) EPSC curves of the albumen-gated synaptic transistor stimulated by gate pulses with the same width of 1000 ms but different amplitudes. (**b**) EPSC retention curves of the albumen-gated synaptic transistor for gate pulses with the same amplitude of 6.0 V but different pulse widths. (**c**) Time-dependent EPSC curve stimulated by six gate pulses (6.0 V, 200 ms) with an interval of 3000 ms. (**d**) Memory decay curves stimulated by different numbers of gate pulses.

In nervous system, transforming of STP to LTP through repeated stimulation is of great significance for long-term memory formation^44^. Here, we investigate the STP to LTP transition in our synaptic devices. The gate pulse voltage is regarded as the external stimulus and the nonvolatile channel current is regarded as the memory level. Fig. 5a shows the EPSC retention curves for gate pulse with the same width of 1000 ms but different amplitudes. No obvious nonvolatile channel current is measured when a gate pulse of 2.0 V is applied. When the gate pulse increases to 4.0–8.0 V, obvious nonvolatile increases of channel current can be observed, and larger gate pulse results in higher memory level. A maximum retention channel current of 101 μA is measured when the pulse voltage is 8.0 V. Fig. 5b shows the EPSC retention curves for gate pulses with the same amplitude of 6.0 V but different pulse widths. When the pulse width is increased from 100 to 1000 ms, the EPSC peak current increases from 13.1 to 145 μA, and the retention current increases from 3.8 to 39.8 μA. So our results demonstrated that longer pulse width would result in a higher memory level, which is similar to that observed in our brain^45^.

The competing effect of memory loss and memory strengthening upon stimulus is also clearly observed (Fig. 5c). The stimuli are set by six gate pulses (6.0 V, 200 ms) with a interval time of 3000 ms. The retention time of the memory state is dependent on the history of previous input pulses. An obvious increase of the channel conductance after each gate pulse and an enhancement of the total current after six gate pulses can be observed. The observed phenomena is similar to the well-studied biological processes referring to that repeated study can result in long-term memory^9^. Notably, not only is the amplitude of the channel conductance increased upon repeated stimulation, the retention time also increases significantly with the pulse number (Fig. 5d). The measurements were performed with the same initial channel conductance and the same gate pulse (6.0 V, 1000 ms). Different number (N = 1, 5, 20 and 40) of gate pulses were applied to the gate electrode and the channel current retention curves were recorded after the last stimuli in each stimulation series. A memory decay of ~80% is observed after 500 s when the pulse number is one. When the pulse number increases to 40, the memory decay is decreased to ~20% after 500 s. Such memory decay curves are similar to the forgetting behavior in psychology. The memory retention of our synaptic device can be greatly improved by increasing the number of gate pulses. These results indicate that a process of rehearsal is important for short-term memory to long-term memory transition, which is similar to that in a biological neural system^44^. The mechanism of synaptic memory emulation in our device is related to the interface electrochemical reaction (hydrogenation), which is in agreement with the above EIS analysis. More protons will penetrate into the IZO channel layer under gate pulse with higher amplitude or more repeated gate pulses, and then surface hydrogenation will result in larger channel current and longer retention time^36^.

**Figure 6:**
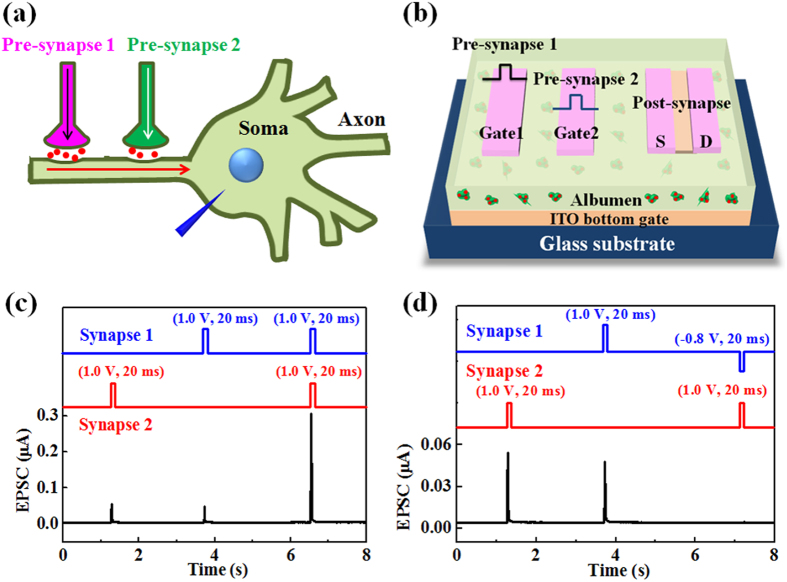
(**a**) A schematic diagram of the synaptic integration from two different pre-synapses (Drawn by Dr. Guodong Wu). (**b**) A schematic diagram of the albumen-gated synaptic transistor with two input terminals (two in-plane gates) (Drawn by Dr. Guodong Wu). (**c**) Supralinear summation of two pre-synaptic spikes. When the pre-synaptic inputs are applied synchronously with voltage from 0.5 to 1.2 V at 0.1 V step, the measured currents are recorded and plotted against the predicted currents. Inset: when two pre-synaptic voltage spikes (0.8 V, 10 ms) are simultaneously applied on G_1_ and G_2_, the predicted current and the measured current are recorded. (**d**) Shunting inhibition. When the inhibitory and excitatory pre-synaptic inputs are stimulated together, almost no response current peak can be observed.

The above synaptic functions illustrate that temporal synaptic plasticity functions (temporal summation). Besides, in our brain, most neurons receive thousands of synaptic inputs, and synaptic spatial summation is one of the basic principles for neuronal computation. Fig. 6a shows the schematic diagram of spatial summation from two different pre-synapses. The spiking signals from two pre-synapses are summed in the dendrite of a post-synaptic neuron. As shown in Fig. 6b, two in-plane gates are used as the two pre-synaptic input terminals and the channel conductance is used as the synaptic weight. As shown in the inset of Fig. 6c, when a pre-synaptic voltage spike (0.8 V, 10 ms) is applied on G_1_, an EPSC peak of 8.8 nA can be measured in the IZO channel layer, as shown by the green curve. When another pre-synaptic input spike (0.8 V, 10 ms) is applied on G_2_, an EPSC peak of 12.5 nA can be obtained, as shown by the red curve. The mathematical summation of the two EPSCs, which is defined as the predicted current, is 21.3 nA (the yellow curve). When two input spikes (0.8 V, 10 ms) are applied on G_1_ and G_2_ simultaneously, as shown by the black curve, the EPSC (the measured current) measured from the IZO channel layer of the device is estimated to be 105.1 nA, which is obviously much higher than the predicted current. When the amplitudes of the pre-synaptic spikes from two inputs are changed from 0.5 to 1.2 V at 0.1 V step synchronously, the measured EPSCs are plotted against the predicted currents. As shown in Fig. 6c, supralinear summation is observed. This result is in good agreement with that observed in biological experiment, and supralinear summation in neurons is of great significance for neuronal computing^46^.

In our brian, some synapses take the membrane potential away from the action potential threshold, and these synapses are called inhibitory synapses. Inhibitory synapses exert a powerful control over a neuron output. In psychology, when the inhibitory and excitatory inputs are stimulated together, the depolarizing current leaks out before it reaches the soma. This phenomenon is called shunting inhibition. In our device, the positive pulse is regarded as the excitatory input and the negative pulse is regarded as the inhibitory input (Fig. 6d). When the excitatory (1.0 V, 20 ms) and inhibitory inputs (−0.8 V, 20 ms) are applied simultaneously, almost no response current peak can be observed. This result is similar to the shunting inhibition in our brain. Input–output gain modulation is critically important for normal sensory and cognitive functions. Shunting inhibition is a longstanding and widely cited candidate mechanism for the gain modulation^47^. Our brain is a very complex nervous system where one synapse is connected with thousands of other synapses, and synaptic spatial integration is much more complicated than our experiments. In the future, we can mimick the spatial summation with more synapses just by introducing more in-plane gates. Therefore, such in-plane multi-gate configuration is very convenient to investigate synaptic spatial information summation.

## Conclusions

In conclusion, proton-conducting natural chicken albumen was proposed as the coupling electrolyte films for the fabrication of oxide-based synaptic devices. Temporal and spatial biological synaptic functions were successfully mimicked in the synaptic transistors gated by chicken albumen. Synaptic plasticity including paired-pulse facilitation, dynamic filtering and short-term to long-term memory transition were successfully mimicked. Moreover, EPSC summation and shunting inhibition were also emulated in a synaptic transistor with two in-plane gates. Our results demonstrated that the albumen-gated synaptic transistors could be promising for building biocompatible synaptic electronics and brain-inspired systems.

## Method

### Materials

ITO glass with the square resistance of 15 Ω/□ were cut into squar pieces of 2.5 cm × 2.5 cm. Albumen liquid was obtained from chicken eggs (purched from the supermarket) using a stainless steel mesh spoon to separate the egg yolk without any subsequent treatment. IZO ceramic target (mass ratio: 90% In_2_O_3_ and 10% ZnO) from China KeTai New Materials Co. Ltd.

### Device Fabrication

For the fabrication of synaptic transistors, conducting ITO glass was used as substrates and bottom gate electrodes. The surface of the ITO glass was cleaned thoroughly by acetone, ethanol, methanol and de-ionized water in sequence and dried by high-purity nitrogen gas. Albumen films with the thickness of 1.0 μm were spun on the cleaned ITO glass substrates at 3000 rpm and dried in air ambient to form a solid film. Then, two designed nickel shadow masks were used to deposit 30 nm indium-zinc-oxide (IZO) channel layers and 100 nm IZO source/drain electrodes on the albumen-coated ITO glass by radio frequency magnetron sputtering at room temperature, respectively. The radio frequency power, Ar flow rate and the chamber pressure were 100 W, 30 sccm and 0.5 Pa, respectively. The channel length (L) and channel width (W) of the synaptic transistors is 80 μm and 1.0 mm, respectively. For leakage current density and electrochemical impedance spectroscopy measurements, ITO/albumen/IZO sandwich structures with a top electrode size of 100 μm × 100 μm were also fabricated.

### Characterizations

The surface morphology and roughness of the albumen film was characterized by AFM using a NanoScope IIIa in tapping mode. Electrochemical impedance spectroscopy (EIS) of albumen film with sandwich structure (ITO/albumen/IZO) was characterized by a Solartron1260A Impedance Analyzer in air ambient. Electrical performance and synaptic behaviors were measured by Keithley 2612B/2636B Source Meter System. Both the complex impedance analysis and electrical characteristics were performed at room temperature in air ambient with a relative humidity of 60%.

## Additional Information

**How to cite this article**: Wu, G. *et al*. Artificial Synaptic Devices Based on Natural Chicken Albumen Coupled Electric-Double-Layer Transistors. *Sci. Rep*. **6**, 23578; doi: 10.1038/srep23578 (2016).
